# Cats in Palace Museum: A narrative of cultural heritage and empathy of youth

**DOI:** 10.3389/fpsyg.2022.1003455

**Published:** 2022-10-13

**Authors:** Yunjiao Guo, Tianle Huang, Qiuju Luo

**Affiliations:** School of Tourism Management, Sun Yat-sen University, Guangzhou, China

**Keywords:** cultural heritage, cats in palace museum, narrative empathy, youths, text analyses

## Abstract

While research in heritage tourism tends to focus on cultural and anthropogenic motivations and drivers, this paper seeks to examine how social-media narration amidst a broader backdrop context in Chinese mass culture creates, perpetuates, and reinforce feline-focused narratives and practices among social media young followers. Drawing on text and image-based analyses of postings of cat sightings within the official Palace Museum account on a key Chinese microblog, this study reveals the application of three vital narrative strategies at work and corresponding empathic responses: ambassadorial, bounded, and broadcast. The Palace Museum has achieved an enhancement of interaction and emotional exchange between the heritage of Palace Museum and youths and generated a process from attention to emotional engagement and eventually to emotional identification on the part of youths in their attitude toward the heritage of Palace Museum through the workings of three key narrative strategies on Chinese social media. In doing so, this research illuminates the potential of social media-based narratives and charismatic animals in the revitalization of cultural heritage sites and the contributions of setting narrative strategies in engaging the younger audiences while also revitalizing the cultural heritage of the Palace Museum.

## Introduction

The Imperial Palace of China, formerly called Forbidden City, is the imperial palace of the Ming and Qing Dynasties. It was listed as a “World Cultural Heritage” site in 1987 by UNESCO and is now commonly known as the “Palace Museum.” The World Heritage Organization describes the Palace Museum as follows: “The Forbidden City had been the center of the highest authority in China for over five centuries. With an architectural complex including garden landscape and 9,000 rooms of furniture and handcrafts, it is a priceless historical testimony to the Chinese civilization of Ming and Qing Dynasties.” However, since the founding of the Palace Museum in 1925, due to incidents of fire and theft, many national treasure objects with high historical and cultural exhibition value had to be mothballed in storerooms for their protection. Yet, it is noteworthy that since Mr. Shan Jixiang became the sixth curator and “gatekeeper” of Palace Museum in 2012, the official stance on the heritage of Palace Museum began to change its formerly detached and aloof style and assume a “populist common touch.” The Palace Museum has adopted a cultural, story-based, and interactive approaches *via* its Sina Microblog and WeChat accounts to promote the culture of the Palace Museum outside of its palace walls to enter into the sight of common people. Further, it endeavored to promote a comprehensive, rejuvenating transition of the Palace Museum, by representing and advertising the Palace Museum in new ways favorable and receptive to the youth.

The rejuvenating transition of the Palace Museum was a resounding success and led to an exponential growth in visitor volume. In 2018 (4 years after the commencement of tourism rejuvenation in 2014), the Palace Museum had become the most visited museum in the world and broke their own annual visitorship record with a staggering 17 m visitations. In the same year, the Palace Museum had also broadened its reach to the general population by expanding its online content to nine different kinds of mobile applications covering a variety of contents from official news, games, and visitor guides. It further cemented its online popularity by integrating its official website with commonly used Chinese applications such as Weibo (China’s equivalent of Facebook) and Weixin (China’s equivalent of WeChat), and put forward a series of new measures to attract younger tourists. These targeted measures proved effective as millennials now comprise the majority of visitors and 40% of visitors are below 30 years old. In the process, the rejuvenation policy succeeds in attracting new groups of younger visitors. Attracting a younger group of domestic tourists is vital to the museum’s long-term sustainability as an attractive tourist attraction.

Especially in today’s China, with a new media environment as represented by microblog and WeChat, not only does the subject of narration demonstrate a pattern of “decentralization,” but also previous narrative strategies and narrative methods of stories depending on traditional media have also changed substantially. The emergence of the Internet and new media platforms has provided new spaces and measures for the dissemination of the heritage. Palace Museum had adopted hundreds of stray cats with an original purpose of eliminating mice for the protection of architecture. But the Palace Museum managed successfully to attract attention and participation from youth visitors by means that appeal to young people, e.g., telling cat stories *via* microblog as an online socializing platform; in the meantime, attraction online drove the tourists’ visits offline and directly triggered a mass fervor to the Palace Museum to see the cats among the youth. Many young people have indicated on social media that their primary purpose of visiting the Palace Museum was to see the cats there. In fact, the operations of empathic narrative strategies stimulate and even sustain the tourist attraction on the narrator’s object (i.e., Palace Museum).

To understand the increased interest in the palace cats and the palace museum, we turn to theories of empathy. However, although the concepts and theories of narrative and empathy in a cross-disciplinary view are well recognized in the field of tourism studies, not only have “how to narrate” and “how to engender empathy” not been effectively applied to the research of heritage protection and utilization, but the idea correlation and functioning between narrative and empathy also have not been sufficiently discussed or explored. By setting a narrative empathy analysis, we examine the interplay between creation and consumption of narrative strategies between producers and consumers and seek to fill this scholarly gap by drawing upon theories of narrative and empathy from an interdisciplinary perspective, to study the stories of the adopted cats in the Palace Museum on Chinese social media.

Therefore, this research adopts the theories of narrative and empathy from an interdisciplinary perspective, targets the stories of cats in the Palace Museum appearing on the microblog social media as the object of case analysis, and discusses with what narrative strategies the narrator used to tell the stories of cats in Palace Museum, and how the cats’ stories and their dissemination have produced an empathic effect on Palace Museum in the mind of youths. Answering the questions above effectively will have an innovative theoretic significance and an instructive practical value for the research of the dissemination, inheritance, vitalization, and utilization of relics of cultural relic sites.

## Literature review

### Tourism and narrative

Narrative originated from literature and linguistics and later entered the domain of geography as a means. The role of narratives in understanding society and societal change is a vital one (Cameron, 2012; Lu, 2013), and various scholars have highlighted the need to connect story and narrative concepts with place and its cultural connotations and discuss their narrative strategies, dissemination methods, and constructive meanings (Blunt and Rose, 1994; Lorimer, 2008). Research on narratives in the field of tourism studies lies in tourism marketing (Stokowski, 2002; Lichrou et al., 2008; Chronis, 2012). For example, Lichrou et al. (2008) maintains that tourism is not simply about places, but is about the experience of a place, about meeting people, and the interactions between hosts and visitors and those with fellow tourists. Narrative is a means through which places are constructed (Stokowski, 2002). Narrative relates to the marketing of place to the consumption experience (Lichrou et al., 2008). Tourist spots with stories can not only present a quality aesthetic experience of the mystery of the tourist destinations and make the cultural connotations and context of the tourist spots easy to understand and perceive, but storytelling can also allow places to be salient destinations and change the attitudes of consumers toward these attractions and entice them to visit (Chronis, 2012); Moreover, from the perspective of consumers’ feelings, research of influences of narrative on the experience and consumption of tourists and humorous storytelling in product advertising can evoke emotional responses to form brand attitude and image (Rhie, 2014), and a good story can stimulate consumers’ interests (Escalas, 1997). It can also create emotional connections between consumers and destinations (Jones et al., 2016), and even transform a place into a high-quality and identifiable tourist spot with aesthetic significance, further change consumers’ attitudes towards a destination and entice them to visit (Wong et al., 2015).

Although the research in the field of tourism have strongly affirmed the positive values of narrative on the tourist industry, so far they have mainly focused on the experience of tourists and the marketing effects of the destinations and touched less on topics such as narrative strategies and methods of narrators. Meanwhile, although there are a few scholars who have stressed that stories have an important value for the formation of cultural identification with the heritage sites from the perspective of urban heritage designers and suggested that shaping a heritage site with a sense of cultural identification requires the tangible material factors to be handled well, as well as addressing the question between the site and its historical stories. These are both essential and indispensable to constructing cultural identification (Frenchman, 2011). Through the weaving a narrative, cultural emotions in the places can be evoked, characteristics and connotations embedded in the places can be interpreted (Lorimer, 2003), generating productivity and participation (Gibson-Graham, 2008). And there is a growing realization that heritage is a process of cultural production, and that the disciplines involved in heritage research and practices are involved themselves in the making of heritage (Smith, 2011). However, research and discussions on introducing narrative theories from a cross-disciplinary perspective and applying them to the protection and utilization of cultural heritages have remained sparse to this day.

### Tourism and empathy

Empathy, a vicarious and spontaneous sharing of affections, can be provoked by witnessing another’s emotional state, by hearing about another’s conditions, or even by reading (Keen, 2006). While Hollan and Throop (2011) and Tucker (2016) position empathy as the sharing of another person’s perspective and unlike other “altruistic emotions” such as sympathy, empathy has an added dimension of “identification with the other (person).” Given the human relational aspects of tourism, empathy inevitably plays a covert role in the construction of experiences in all contexts. Adaval et al. (2007) remind us that empathy is a decisive influence over individuals’ attitudes and behaviors when engaging in tourism activities. Empathy, as a social science concept has long been explored alongside tourism studies in areas including but not exclusive to tourists’ experience (Adaval et al., 2007; Andermann and Arnold-de Simine, 2012), volunteer tourism (Pedwell, 2012; Butcher, 2014), tour guiding and interpretation at museums and heritage sites(Dimache et al., 2017), ‘dark tourism’ (Miles, 2002; Stone, 2006), tourism and the environment, tourists’ ethics and behavior (Norridge, 2009), tourism and hospitality (Barlow and Maul, 2000), and so on. Of particular interest is the idea of “historical or heritage empathy.” Visitors experience being concerned for people in the past and how they experienced their lives (Barton and Levstik, 2004) and tourists on these tours are encouraged to “adopt cognitively a perspective different from their own and to establish an emotional connection with historical actors from different eras and walks of life” (Modlin et al., 2011). Selected researchers in “dark tourism” also discovered that visitations to macabre sites where actual death and suffering took place (e.g., Auschwitz) evoke a stronger empathic response from visitors more so than museums of the same theme (Miles, 2002; Stone, 2006).

To date, tourism studies have mostly focused on the “results” of empathy, i.e., positive effects of the tourists’ empathetic responses on their experience of touring destinations; of particular interest is the experience of being there among “others” (Tucker, 2016). However, there is a noticeable literature gap in the study of the connection of empathy to heritage narration in tourism studies (i.e., what causes empathy, how empathy is processed and what strategies are used to evoke empathy), especially narrative empathy. Although stressing the formation of historical empathy in the research of historical and cultural heritages is conducive to the evocation of the emotional resonations of tourists, research that focuses on the means and methods to evoke tourists’ empathetic emotions have been insufficient.

Gallagher (2012) suggests that “narrative” is a fundamental mechanism of means in the arousing of “empathy” in individuals. Nevertheless, the application of the two has been separated and there was little research on integrating “narrative” and “empathy” to present them together. For example, how do the narrators’ narratives of destinations trigger the empathetic responses from tourists or potential tourists? There was especially minimal progress in the research with youth as the main target of research that could be applied to the utilization and vitalization of cultural heritages.

## Research method

### Theoretical framework

This study adopts the narrative empathy theory (Keen, 2006) as its theoretic framework. As a cross-disciplinary theory, the narrative empathy theory involves multiple domains, such as literary narrative, cognitive narrative, theory of reader response, marketing, and contemporary neuroscience. It refers to the emotional resonation (integration) of readers with text and characters in the story, including alternative situation in reading, checking, listening, or imagining stories and condition-induced shared sentiments and empathetic understanding (Zhong, 2017). The narrative empathy theory mainly focuses on the two aspects of “narrative” and “empathy.” Its concerned targets are the psychology and emotion of authors or readers, and its purpose is to reveal how various narrative techniques evoke empathetic responses from readers. As narrative mainly includes three factors, i.e., narrator, audience, and narrative’s object. On the online platforms, the storyteller(s) of these “cat stories” on the Palace Museum’s official online platforms play(s) the role of the “narrator,” with the palace cats often forming its central objects of interest, set within photogenic backdrops of imperial Chinese heritage, and the followers of this official account are the “audience.” We seek to add theoretically to the understandings of the operations of empathic narrative strategies by unpacking the entanglement between these three elements.

The specific theoretic framework of this study consists in the three strategic narrative empathies (Keen, 2006), which are bounded strategic empathy, ambassadorial strategic empathy, and broadcast strategic empathy. Keen (2006) maintains that all three strategic narrative empathies are narrative strategies and techniques adopted by creators/publishers to influence or even manipulate the emotions, attitude, and behavior of targeted audiences. In other words, most authors attempt to evoke attention, empathy, and identification in readers to realize certain purposes. Thus, this also corresponds to the question to be answered by this study, which asks, how has the Palace Museum managed to build an active interactive relationship between people and cultural heritage sites and evoke an empathetic response from the audience through the cat stories? Here, we will build on the narrative empathy theory to examine how adopted palace cats and social media come together to shape and reshape tourism at the Palace Museum, an official and formal cultural heritage site.

### Methodology

In terms of research method, this study mainly adopts a method that combines quantitative narrative analysis and qualitative narrative analysis. As a means of sociological research, narrative analysis effectively integrates a variety of single or synthesized research, such as definition questions, descriptive questions, process questions, oral interactions, dialogue questions, and behavioral questions (Wang, 2013). Most narrative or story studies, particularly in narrative transportation experiments, exclusively focus on traditional written texts (Van Laer et al., 2014). However, narrative is ubiquitous in the sense that narrative in the form of text can include words, pictures, music, novels, and advertisements, and the audience of stories can be a reader, spectator, listener, and even a tourist.

This study purposefully selected “*Gugong Baidianer*” (*Palace Museum White Dot*), as our site of analysis. *Baidianer* (hereainfter White Dot) was the name of a cute and furry (deceased) cat in the museum. However, many other furry cats, such as Aobai, Xiangcai, Xiaoliu, and Changtui are also featured on the microblog. This account has a large audience with more than 110,000 active followers. Since material in this microblog is mainly presented through a combination of text and pictures, we utilized two methods to analyze the content for our study purposes. All the text-based and photographic material quoted and referenced in this paper are from this official microblog unless otherwise stated.

Firstly, this study used the octopus web crawler software to collect the text contents of 176 entries posted between June 30, 2018, and April 12, 2019, and obtained a collated sample document to be used for the acquisition of high-frequency words in the microblog.

Secondly, pictorial analysis was used to conduct a narrative analysis of the pictorial texts, through analyzing the respective proportions of elements of the Palace Museum and the cat elements in the pictures vis-à-vis the total size of the pictures, to reveal the unique identity construction of the cats and their followers in the curated pictures. The selection of pictures was done through random sampling (*n* = 452) of pictures published before April 12, 2019, in the photo album aptly named, “White Dot of Palace Museum.” We followed three criteria in our selection: individualized; clear and easily identifiable, and no duplicate pictures. After applying these three criteria, we obtained 442 qualified pictures from which a further 100 pictures are selected randomly as samples for analysis. In the proportional analysis of pictorial elements, we adopted method of Lu and Song (2016) of analyzing the composition of pictorial elements in the classic garden landscape plane graph and used AUTOCAD 2019 to mark out the elements in the sample pictures and calculated the areas and proportions of the composing elements in all the 100 pictures, respectively.

## Study findings

### Ambassadorial strategic empathy: Evoking attention of audiences with construction of the “special identity” of cats

Narrators who utilize ambassadorial strategic empathy first attract the attention of readers and subsequently cultivate and sustain readers’ interest through playing up the uniqueness of the narrated object of interest (Keen, 2006). In our study, firstly, narration alongside curated pictures of the palace felines reinforces the identity of the royal cats by highlighting distinctive elements of the Palace Museum; these curated pictures possess sharp visual perceptibility and strong aesthetic characters. Secondly, text narrative as caption of the pictures has not only a flavor of the imperial court, but also a strong characteristic of personification. This narrative strategy has intensified the construction of the special identity of the cats and speaks to the readers. These cats are no ordinary cats but court cats living in the imperial palace. They are unique and nowhere else to be found. The narrator of stories of the cats in the Palace Museum adopts a method of combining pictures and text and publishes cat stories through the microblog account. According to the theory of Narratology, relating and representing scenes of cat life in the Palace Museum is a narrative in nature. Therefore, this study first conducts a qualitative narrative analysis of the pictures and text of the microblog account, “White Dot of Palace Museum.”

#### Analysis of pictorial narrative factors: Highlighting elements of palace museum under the theme of cats

To reveal the reasons behind the attention paid by youths to the cats in the Palace Museum, we marked the various elements in the 100 samples with AUTOCAD software, calculated a proportion of each pictorial element in each picture, and obtained the average area of each pictorial element. The results are shown in Table 1.

**Table 1:** Average proportions of composing elements in the 100 pictures.

Elements	Cats	Palace Museum				Others
Content	Cats	Red/white wall (8.01%)	Red window (4.05%)	Roof and tile (3.26%)	Floor (30.66%)	Sky, flying birds, and bicycles
		Flower, grass and tree (7.32%)	Red gate (1.84%)	Other elements of Palace Museum (Long chairs, antiques, rockery, columns, etc.) (3.20%)		
Average proportions in the pictures	22.49%	58.34%				19.17%

It can be seen from Table 1 that the various elements in the Palace Museum such as the palace’s distinctive, red-themed architecture such as doors and windows, flowers, and trees et al. occupy a significant area in the pictures, and their combined total proportion far exceeds the cats. As distinctive cultural symbols representing architecture of the Palace Museum, such as the red wall and red gate, etc., are consciously highlighted. Lively cats shuttle in the space amid historical architectures, with the dynamic animal and static architecture complementing each other in representation. The depictions of the daily life state of the cats in the pictures have in effect shortened the distance between the architecture in the Palace Museum and in real life, while the distinctive background symbols in the Palace Museum have unnoticeably constructed the special aesthetic effect of the palace cats.

The micro-blogger, i.e., the recorder and narrator of the life of the cats in the Palace Museum, has masterly integrated elements of the cats with that of the Palace Museum through the composition design of photographing, which not only enabled a strong aesthetic connection visually between the cats and the Palace Museum, but also in the meantime conferred on the cats an extremely strong specific identity of “cats of Palace Museum”. The specificity of the composition and narrative of these pictures and the aesthetic sense they engender have evoked the empathetic response from readers, and the empathetic effects are mainly demonstrated as follows: first, the story readers change their degree of involvement from looking at, to caring about, and choose to become fans of the microblog account “White Dot of Palace Museum” with comments such as “having become follower of the White Dot account,” “the 36th fan of the White Dot is here to report,” “seeing the stories of the cats of Palace Museum, I became a follower right away,” etc.; second, after receiving the information on the identity of the cats implied in the pictures, readers show their affection and appreciation of the cats by emphasizing the specific identity of “Palace cats” in terms of cognitive and emotional expressions with comments such as, “all cats in the Palace Museum are so cute,” “cats of the Palace really look pretty,” and “cats certainly match better with Palace Museum”; finally, having gained such an aesthetic experience through the pictures and stories of “Cats and Palace Museum,” readers tend to shift their acknowledgement and praise from the stories to the narrator of the stories, which incurs a “from-animal-to-human” empathetic effect, exemplified by comments such as, “what a lovely photographer that could produce such photos!” and “falling in love with the photographer of Palace Museum.”

#### An analysis of word frequencies of the caption texts: Aulic tendency and personification of cats

We also analyzed the content of the texts by using the analytical functions of social networking and the semantic network of RostCM6 software. Words appearing more than 10 times were selected. Those considered meaningless or irrelevant to the cats in Palace Museum were excluded. The results are shown in Table 2.

**Table 2:** List of high-frequency words and the word frequencies in the microblog text.

Serial number	Keywords	Word frequencies	Serial number	Keywords	Word frequencies
1	Palace Museum	71	22	Elder brother	16
2	White Dot	69	23	Wander	15
3	Coriander	42	24	Uncle	15
4	Friend	38	25	Mom	14
5	Elder sister	34	26	Hospital	14
6	Beijing	30	27	Ci Ning Palace	14
7	Pussy	27	28	Look after	13
8	Black Tail	25	29	Photographer	12
9	Copycat	25	30	Master	12
10	Doctor	24	31	Pet	12
11	Adoption	23	32	Kitten	12
12	Story	23	33	Name	12
13	Museum	23	34	Thank	11
14	Colleague	22	35	Thirty	11
15	In the yard	21	36	Surgery	11
16	Younger sister	20	37	Question	10
17	Empress dowager	19	38	Time	10
18	Sterilize	18	39	Audience	10
19	Three-Colored	18	40	Child	10
20	Tin can	18	41	Princess	10
21	Baby	17			

After analyzing the word frequencies, it was discovered that “Palace Museum” and “White Dot” have the highest frequencies, and the former slightly more frequently than the name of the cat. This demonstrates that the narrator has used the linguistic symbol, “Palace Museum” frequently in his/her literal narrative. In other words, in the storytelling of “cats” and “Palace Museum,” the portrayal of the character of the cat “White Dot” as the protagonist has always been placed in the historical space and time of the Palace Museum; second, viewed from the part of speech, the frequently appearing words in the tales of the microblog account, “White Dot of Palace Museum” are mainly nouns and mostly appellations, such as “empress dowager,” “master,” “princess,” “younger sister,” “elder brother,” etc., and especially many appellations that match the historical scenes of the Palace Museum like “empress dowager,” “master,” and “princess,” which not only personifies the cats, but in the meantime also connect the cats with the historical scenes and culture of the Palace Museum. Moreover, these historical appellations have not only made the image of the animals interesting, but also put readers in the historical scenes of the Palace Museum.

The key for the occurrence of narrative empathy consists in the choice of perspectives on the basis of space. As a result, readers have produced two kinds of empathetic responses. One is that the readers shift from the current space and time to the historical space and time, personify cats to imply human nature, and adopt utterances of the imperial court to interact with the cat stories. Examples of this include comments such as, “wish your majesty (meaning the cat) eternal health and felicity,” “I (meaning the cat) sit up in the palace,” and “blessings to the empress dowager.” The other response is that readers cosplay the figures of the imperial palace in history through the cats. This reinforces the representation of historical scenes in the imperial palace and the imagination of historical figures in the cats’ tones, such as “Aobai (name of a cat) said: ‘quite a few people are coming to court lately,’” “This is Aobai, present yourselves before me!,” and “Aobai (name of a cat) said: ‘I cannot bow my head, because My crown might fall off.’”

### Bounded strategic empathy: Evoking the emotional engagement of audience from multi-narrative perspective

Keen (2006) points out that Bounded Strategic Empathy mainly targets members of a group and galvanizes readers into producing certain emotions toward those with whom they are familiar based on common experiences. This particular narrative strategy is characterized by telling stories with features of similarity to or familiarity with readers in terms of the story content to pull the readers closer to the stories. The generation of responses to Bounded Strategic Empathy usually requires a comprehensive application of multi-narrative perspective, and seeks to apply a variety of narrative perspectives in the storytelling technique to enter the inner world of different readers and enhance their emotional engagement.

#### Narrative strategy of internal perspective: Evoking the empathetic response “from animal to human”

Keen (2006) believes that the internal perspective mainly consists of three types, namely narrated monologue, interior monologue, and psychonarration. In the microblog stories of the cats in the Palace Museum, the narrator conducts a fully illustrated representation of the characters and images of the cat. The texts usually adopt a narrative strategy of an internal perspective, and use a first person “I” to depict the mental activities of the cats. The first person narration and the psychological description in representation of the characters’ consciousness and emotions can help readers identify with the characters and facilitate the evocation of empathetic experience (Keen, 2006).

In Type 1 illustrated narrative of “narrated monologue,” the author takes a picture of a cat sleeping in the Palace Museum and accompanies it with descriptive text. The life of the cat recorded tallies with the pace of life of human beings, and readers can discover a sense of familiarity and common ground in the life and mental state of the cat. In type 2 illustrated narrative of “interior monologue,” the author captures changes in the movement and manner of the cat and uses apposite words to directly demonstrate the interior monologue of the cat from being commanding to meek and then to helpless. The three pictures and their captions render a delicate and vivid portrayal of the cat’s character and present the mental activity of the cat as a fluid, fickle, and corresponsive process, which facilitates the understanding and adds more enjoyment on the part of the readers. In type 3 illustrated narrative of “psychonarration,” the author demonstrates a story of dialogue between different cats with text, which enriches the originally monotonous life of cats, projects the course of human interaction on the life of the cats, and renders a sense of intimacy to the readers.

Through the narrative strategy of internal perspective, the story narration will possess a strong sense of empathy and authenticity. After reading the stories successively, readers develop a sense of familiarity with the cats, and also forge an emotional relationship between themselves (as cat observers) and the cats. The readers thus have progressed gradually from initial observation to participation and voluntarily increased their emotional engagement by immersing themselves in the world of cat stories, which begets the effect of immersion, which are demonstrated by the readers’ comments such as “*Miss you, White Dot,*” “*Thinking about the White Dot everyday,*” “*Waiting for the renewal of the microblog stories everyday,*” “*Paying attention to the White Dot everyday,*” “*Really missing the White Dot and expecting to hear your stories about the cats in Palace Museum,*” etc. Meanwhile, empathetic responses generated by this narrative strategy are mainly shown as being an “animal-to-human” process: Firstly, readers associate cats with themselves, project the living conditions of the cats on their own state of life, and express their longing for the free-living cats, exemplified by comments such as, “*How I wish myself being a cat and lying there quietly and leisurely, without having anything, good or bad, to worry about. Being free is all I want,*” etc. Secondly, readers associate the cat with the staffs of the Palace Museum, and through the cats, emotions of respecting and praising those who take care of it are evoked, as manifested in comments such as “*In fact, I am most grateful to you guys (staffs of Palace Museum). As a national cultural unit, you are now a protector of stray cats, which not only sets up a shining example for others, but also helps foster a good idea among the public that humans and animals can always live in harmony with each other,*” “*I believe that as a fine model of loving cats, staffs of Palace Museum can play a huge role on protecting poor stray animals and creating a harmonious cultural environment. Thank you for your effort,*” etc. Lastly, readers associate the cats with human beings in general and a reflection on human ethics and emotions is evoked through the character and qualities of the cat in the stories, which are displayed by comments such as, “*Perhaps on White Dot, sometimes we can find what we humans lack, like the desire to share all the happiness with others, such a sincerity and selflessness,*” “*Being a human or a cat, one needs to be the simple and honest kind, to be respected by others,”* and “*We humans really need to have a loving heart.*”

#### Narrative strategy of external perspective: Evoking the empathetic response “from animal to place”

“Internal perspective” and “external perspective” are opposite outlooks of each other. The difference between the two consists in whether the observing position of the story narrator is “inside” or “outside” the “story” (Zeng, 2005). External perspective is in fact a knowledge-limited perspective, which can be demonstrated by the formula “narrator<figure” of Genette (1990). Due to the limitations of the observing angle of the narrator, readers can only see the behavior of the figure in action from the perspective of the narrator but have no idea of the emotion and mental activities of the figure; while the external perspective can also directly guide readers to form a value concept over certain events through outlining the background of the story (Jiang and Zhang, 2018). The narrative strategy of external perspective narrates cat stories from the third-person narrative perspective, maintains the position of an observer and does not interfere with the mental activities of cats. Such a perspective can trigger more space of imagination and judgment of readers over stories and cats as the protagonist of the stories. In the examples, such as “*Next time when touring Palace Museum, I shall be as curious as a cat, opening my cat’s eyes wide and erecting my cat’s ears, to observe and feel about this city with my heart.*” Another narrator makes an objective recording of the life of the palace cats, by commenting: “*This weekend at the Ci Ning Palace (one of the palaces in Palace Museum), you cannot entice Coriander (name of a cat) even with dried fish. She has just received a de-worming vaccination shot and is being kept for monitoring. You guys may want to take more look at the deer in the garden of Ci Ning Palace and the fish at Linxi Pavilion, both having a long history.*” In this comment, the narrator does not speculate or depict the mental activities of the cats, yet the scene of Palace Museum has never been absent, always fostering a sense of specific locality with information of spots such as “Beijing,” “this city,” “Ci Ning Palace,” and “Linxi Pavilion,” which has evoked the imagination of readers over the heritage of Palace Museum and its environment and, through indirect information introduction and impartment, enabled readers to obtain a perception of the geography and environment of the Palace Museum in a subtle and subconscious fashion and gradually created a sense of familiarity between the people and the place.

Through the narrative strategy of external perspective, the narrator attaches a sense of openness and productivity to the cat stories, and readers beget a “from animal to place” empathetic effect under the open narrative approach. Firstly, readers associate cats with Palace Museum and develop an interest in its history and culture, reflected by comments such as “*Thanks to White Dot for making me experience a different Palace Museum,*” and “*One day I shall go to Beijing, to look at the city that has shouldered so much history and look at the red walls and green tiles of Palace Museum.*” Secondly, readers rediscover the value of Palace Museum through cats and engender a warm and tender aesthetic appreciation of the image of Palace Museum, expressed by comments such as, “*Neither can Palace Museum live without these cats, nor can the cats live without Palace Museum,*” “*There is kindness and warmth in the Palace Museum,*” and “*Palace Museum is not just a museum but also a place full of humanistic care and love,*” Lastly, from their positive perception of Palace Museum, readers cultivate spatially a sense of identification with Beijing where Palace Museum is located and the Chinese civilization at large, as demonstrated by comments such as “*Because of the Palace cats, I came to feel that Palace Museum is no longer just about icy cold architecture and relics but a warm and lively place with flesh and blood. Even Beijing has gained some warmth from them,*” and “*The greatness of Palace Museum not only lies in its historical deposits, but all the more lies in its compassion and love for living creatures. May the love be carried on and extended beyond the walls of Palace Museum.*”

### Broadcast strategic empathy: Creating audience’s identification through common life narrative

Keen (2006) points out that broadcast strategic empathy appeals to every reader to emotionally identify with the object of the narrative by stressing common human experiences, emotions, hopes, and fragilities. Broadcast strategic empathy is mainly characterized by narrating stories based on emphasis of human commonality and intercommunity and does not merely present details or relay facts, but also to evoke identification of readers. Under such narrative strategy, readers come to form an understanding of and identification with the cats through caring about and participating in the life experiences of the cats in the stories and gradually develop an emotional identification with the staffs and the cultural heritage of Palace Museum.

#### Topics of broadcast narrative and expressions of audience identification

It was discovered through the research that while cats are the protagonist in the microblog stories, the narrator does not portray the cats or depict their characters elaborately, and the identification of readers with the cats only comes from tiny elements in the stories involving the identity, status, and feelings of the cats. In thus, we selected 15 high-profile stories with three criteria, namely 150 words or more in story length, more than 1,000 likes, and more than 100 comments from the microblog as our objects of analysis. This then formed the basis to produce human themes as shown in (Table 3) the statistical results below:

**Table 3:** Themes in 15 stories.

S/N	Universal human needs/values	Themes in narrative content	No of Likes and %/total comments
Story 1	Connection (with others)	(Love) Sick cat being helped and cared for by volunteers	9,909; 71%
Story 2		(Friendship) Blossoming friendship between two cats	1,953; 81%
Story 3		(Compassion) A tale of kindness and sincerity of cats and appeal for caring about stray cats	864; 87%
Story 4		(Compassion) Sick cat being helped by kindhearted people	1,018; 81%
Story 5		(Romance) Palace cats who found love with each other	822; 81%
Story 6		(Companionship) Interaction between a Palace cat and tourists	799; 78%
Story 7		(Maternal love) A mother’s great love exhibited by a mother cat	621; 74%
Story 8		(Compassion) Adopted cat by museum staff	720; 75%
Story 9	Well-Being	(Shelter) Welcoming a adopted cat to its new cozy home	1,659; 86%
Story 10		(Health) Caring for cats’ health through physical examination	1,985; 75%
Story 11		(Safety and security) The prodigal cat who left and returned to the Palace	6,605; 87%
Story 12	Birthing	(New life) Pregnant cat giving birth	1,149; 81%
Story 13	Altruism	(Sacrifice) Adoption of cat by staff after becoming sick	653; 78%
Story 14		(Sacrifice) Seriously ill cat being looked after by a group of volunteers	462; 75%
Story 15	Death	(Passing of life) Death of seriously ill/elderly cats	461; 80%

Meanwhile, it was also discovered, through counting the feedback information on the stories from readers in the comment section of the 15 stories that firstly, the numbers of likes and forwards of each story accounted for more than 70% of the total number of comments. Actions of giving likes and forwarding directly reflect the positive emotion and attitude of readers toward the stories and are typical practices of expressing approval by the readers. Secondly, besides giving likes and forwarding stories, readers also express their emotions through text comments, which mainly consist of three types: encouragement; expectation; and gratitude.

Text comments from readers with “encouragement” as an expression of emotions are mainly composed of two types. One type stresses the “Palace Museum” identity of the cats and identifies with the strong vitality and the resilient character of the cats. Some examples of this includes comments such as, “*White Dot, you must get better! You are a cat of Palace Museum,*” “*Come on White Dot! You are the most adorable cat of the Imperial Palace*,” The other uses the “affirmative power of human love” as encouragement for the cats and endorses the emotional bond between cats and humans, such as “*Cheer up White Dot, so many people love you, and life on earth is worth living,*” “*Get better soon, we will see you again in Palace Museum,*” Text comments from readers with “expectation” as an expression of identifications are mainly demonstrated in two aspects: one is the expectation to visit Palace Museum as a result of identifying with the cats, such as, “*Cheer up White Dot, I expect to visit Palace Museum to feed you your favorite canned meat after you recover,*” “*You must get better! We will go to see you at Palace Museum,*”; the other is the action of caring resulting from identification with the cats, such as “*Want to know what Palace cats need lately (brands of cat food, snacks), and to what address shall I write if I want to send stuffs to the Palace cats?*” Text content from readers with “gratitude” as an expression of identifications are mainly demonstrated in two aspects: one is the generation of the feelings of identification with and gratitude to Palace Museum and its staffs through the narrator’s detailed descriptions of the cats living in Palace Museum receiving love and care, such as, “*Thank you (staffs and volunteers of Palace Museum) for looking after White Dot*” and “*Thanks to the staffs of Palace Museum for earnestly caring and treating the cats*”; the other is the generation of collective cohesion and the spirit of love among readers from their identification with the practice of Palace Museum and its staffs, such as, “*Please tell me what I can do for it?*” “*We can also dedicate our share of power of love to these cats,*” and “*If there’s need, I am willing to dedicate my humble strength to the medical treatment.*”

#### Empathetic behavior based on emotional identification

Keen maintains that identification (which occurs in readers not texts) is not narrative strategy in itself, but a result of reading, which is probably caused by using a specific strategy, while identification is always likely to evoke empathy (Keen, 2006). Experiencing empathy and imagination may affect the audience’s emotions, cognition, and beliefs, which can further influence attitudes, intentions, and behaviors (Adaval et al., 2007). In the context of this study, through practices of bringing the scenes of the Palace Museum where the Palace cats live into the stories and portray life in the Palace Museum, readers’ empathy for the “Palace Museum cats” has triggered changes in their cognition of “Palace Museum” and behaviors of visiting it.

Firstly, based on narratives of daily life common to humanity, micro bloggers authentically record the life of cats in Palace Museum and transform Palace Museum from a mysterious, cold, and solemn space of cultural heritage to a human-place interactive space that is touchable, palpable, and has a touch of human feeling and warmth. Not only has the youthful audience expressed their love for and identification with the cats online, their attitudes and emotions toward Palace Museum have changed from a sense of distance previously to a sense of intimacy to the heritage itself, and assumed a strong voluntary consciousness to disseminate and inherit it, as displayed by comments such as, “*This change (of Palace Museum) is heartwarming, kitties are being taken good care of,*” “*My great Palace Museum is really full of love! This is exactly how humans and animals should live side by side!*” Secondly, the identification and empathy of youths with the cats in Palace Museum have produced a positive influence on the group behavior of youths. The youth group not only regard the cats as a dynamic intermediary for establishing a real interactive relationship with Palace Museum and as an object upon which to place and express positive inner emotions, but also to find expression of such emotions of identification with the Palace cats in their own daily life behaviors and actions. Shan Jixiang, the sixth curator of Palace Museum, remarked that every day, the Palace Museum receives cat food sent from people across the country from the Northeast to the South of China, some of which even stipulate recipients specifically such as the cats of Ci Ning Palace or Yan Xi Palace. Lastly, the interactive process evoked by the intermediary of stories generated a positive influence on the youth visitors regarding their perception of and sentiment about the cultural heritage of Palace Museum. Furthermore, the cats in Palace Museum have also become a special attraction to young people at the site of this cultural heritage, which are demonstrated by comments of many microblog users, such as “*My little daughter at such a young age is pleading me to take her to Palace Museum, all because of the cats,*” “*Where are cats roaming (in Palace Museum)? Think about buying a ticket to go there tomorrow for the specific purpose to play with the cats,*” “*Since becoming a follower of the microblog, I’ve always longed to get a chance to see those lovely Palace cats,*” and “*For the cats, I’ll go to Palace Museum one more time.*”

## Discussion

Heritages are in essence a process of cultural production that produces meanings. Meanwhile, different social groups have diversified ways of understanding and utilizing heritages (Smith, 2011). Through our research, Palace Museum creatively interprets its cultural connotations and proactively engages the interest of youthful audiences in an environment of new media communication. The ways of narrating Palace cat stories cater to the characteristics of self-expression and free communication of youth groups and fit in with the aesthetic taste of modern youthful culture. Youth groups have developed an attention to and a fondness of the cultural heritage of Palace Museum through viewing pictures and reading stories of Palace cats on social media such as *via* microblog sites, which demonstrate not only a new way of creating the emotional response of tourists (Jelinčić et al., 2021) between the youth groups and heritage sites in an era of new communication, but also the reinterpretation and meaning production of the value of cultural heritages by youth groups. In the meantime, “Authorized Heritage Discourse” holds that heritages are fragile, limited, and non-renewable, and must be protected by experts, who refer to archaeologists, museum curators, architects etc., who are naturally considered guardians of the past and who can understand the value of heritages and convey it to audiences, both domestic and worldwide. Smith (2011) holds the opposite view and believes that not only are heritages important, dynamic, fluid, and not locked in physical forms, but their visitors are not passive either. As such, visitors to museums are users of the heritages, who strive to understand and utilize the heritage productions in multiple ways. Our research effectively verifies views of Smith, and also complements the views on the dynamics of the heritages themselves and the initiative of visitors to a certain degree.

Driven by the world heritage movement of today, the protection, utilization and research of cultural heritages are being converted from a historical venue controlled by elites top-down, to a space that gives access to common people and plays to the value of the heritage’s bottom-up. In essence, heritages are constructed (Zhang, 2008), and constructed by those who are commanders and narrators of the rights of speech (Zhou and Zhang, 2019). The value of cultural heritages is not just locked inside the relics, nor relies on the traditional method of experts’ interpretations only for dissemination. Daily life-oriented construction of the heritage sites does not lower the class of the cultural heritages or decrease their value; on the contrary, the process of “disenchantment” in which the heritage sites move to the visual field of popular aesthetics can actually cause the audience to recast their attention to the heritages and to positively interpret and actively disseminate their value. Although the heritage sites as historical places are detached from their historical environment, through employment of the Internet and new media technologies, they can enter the popular vision assisted by multiple methods of narration and dissemination, activate an emotional resonation of contemporary people. To this end, a salient theoretical point we uncovered here is how animals are featured in the promotion and shaping of a cultural heritage tourism experience at a major Chinese historic site. The ways in which the cats are enrolled into the narration of cultural heritage and its tourism practices resonate with contributions in Actor-Network Theory concerning the re-centreing of animals in our social sciences.

## Conclusion

In a new environment of mass communication, Palace Museum opens the microblog account, “White Dot of Palace Museum” on the microblog platform and endeavors to create a vivid personified image of cats by truthfully documenting the life of the Palace cats through a combination of picture and text and with multiple narrative strategies and methods, which not only has evoked empathetic responses from readers along the attention-engagement-identification line, but also generated in readers an empathetic effect of “identification with animal (cats) to that of human” and “identification with animal (cats) to that of place (Palace Museum).”

Firstly, the narrator employs ambassadorial narrative strategy as the main method of drawing the attention of audiences. The narrator has highlighted and reinforced the symbol of Palace Museum in both pictorial and text narratives by active construction, created an aesthetic relationship between the cats and Palace Museum, and induced the attention of the audience to the Palace cats successfully through constructing a special “Palace Museum” identity of the cats. Secondly, on the basis of successfully induced attention of the readers, the narrator uses bounded narrative strategy as the main method of stimulating readers to increase their emotional engagement and narrate the life of Palace cats through narratives of internal and external perspectives, respectively. Lastly, the broadcast narrative strategy uses narrations based on the daily life of cats to effectively evoke a sense of identification of the readers with the cats through selecting narrative topics that are commonly shared by humans and cats, in which the cat stories become an intermediary of creating a positive emotional bond between the youthful audience and the heritage site, and the Palace cats become a special attraction to young people at the site of Palace Museum cultural heritage.

Palace Museum uses the narration and dissemination of the cat stories as an effective means of reconstructing the modern value of Palace Museum heritage and uses multiple narrative strategies and methods to create positive empathetic responses and effects among youth groups, which has not only attracted the attention to and participation in Palace Museum and its heritage from youths again, but created an identification of youths with the cultural heritage of the Palace Museum. The narration of stories not only can create special attractors for sites of cultural heritage, but also reconstruct the cultural value of cultural heritages so as to ensure the latter is protected and utilized in the course of dynamic narration and active dissemination.

Given that the focus of our image content analysis is focused on the respective proportion of elements of the Palace Museum and the cat elements in the pictures vis-à-vis the total size of the pictures to reveal the unique identity construction of the cats and their followers in the curated pictures. Yet, as emotional arousal of each individual may be different, which depends on various elements (e.g., form, colors, and abstract designs) which influence their empathy level (Jelinčić and Šveb, 2021). Therefore, although our research focus is relatively specific and contextualised compared to general pictorial analysis, manual processing methods have drawbacks in terms of strong subjectivity, complex identification process and significant result deviation. Future studies may use software such as YOLOV2、Google Cloud Visio and Clarifai among others to identify and visualize content characteristics, including culture, architecture or color (Deng and Li, 2018; Jelinčić and Šveb, 2021) to uncover specific visual factors which may influence the narrative and visitor’s involvement, immersion and emotional responses.

## Author’s note

Images used for pictoral analysis will be provided by the authors upon request.

## Data availability statement

The original contributions presented in the study are included in the article/supplementary material, further inquiries can be directed to the corresponding author.

## Author contributions

YG undertook most writing of preliminary draft and the final version of the paper, proposed the theoretical framework of the paper, and also undertook the data collection and qualitative analysis of the most reviews material. QL, who is the YG's postdoctoral advisor, gave a lot of important suggestions for the whole paper, and revised the sections of introduction and discussion of the paper. TH undertook part of data collection and quantitative analysis and also the preliminary draft of literature review, and did part of editing. All authors contributed to the article and approved the submitted version.

## Funding

This study was supported by grants from the National Natural Science Foundation of China (to YG; No. 19CGL033) and the National Science Foundation of China (to QL; No. 41971176).

## Conflict of interest

The authors declare that the research was conducted in the absence of any commercial or financial relationships that could be construed as a potential conflict of interest.

## Publisher’s note

All claims expressed in this article are solely those of the authors and do not necessarily represent those of their affiliated organizations, or those of the publisher, the editors and the reviewers. Any product that may be evaluated in this article, or claim that may be made by its manufacturer, is not guaranteed or endorsed by the publisher.
